# Psychological Wellbeing and Healthy Aging: Focus on Telomeres

**DOI:** 10.3390/geriatrics4010025

**Published:** 2019-02-23

**Authors:** Mariangela Boccardi, Virginia Boccardi

**Affiliations:** 1 Department of Psychiatry, University of Campania Luigi Vanvitelli, 80138 Naples, Italy; mariangela.boccardi@email.it; 2 Section of Gerontology and Geriatrics, Santa Maria della Misericordia Hospital, 60132 Perugia, Italy

**Keywords:** wellbeing, aging, telomeres, stress, longevity

## Abstract

Stress and depression are known to modulate the aging process, and might also affect telomere biology. In fact, exposure to some biochemical pathways involved in stress-related depression may contribute to an ‘‘accelerated aging” phenotype, as well as the incidence of age-related diseases, including metabolic disorders and dementia. Basic studies support the notion that the telomere and telomerase system plays a pivotal role in the aging process and disease promotion. Interestingly, short and dysfunctional telomeres are associated with reduced lifespan, as shown in animal models. In this context, telomeres are very sensitive to stress, mindset, and lifestyle, and their rescue may be sufficient to restore cell and organism viability. This mini-review discusses conceptual models of healthy and active aging and their relationship with telomere biology and mental health.

## 1. Introduction

The total population in Europe is projected to increase from 511 million in 2016 to 520 million in 2070 [1]. Population aging is undoubtedly a demographic success, driven by many changes in fertility and mortality, as well as social and economic developments [2]. However, while life expectancy is continuously rising, the health span (defined as the years of life lived in health) has stagnated for years [3]. If the population has become more long-lived on the one hand, on the other, aging has promoted a progressively higher prevalence of chronic age-related conditions and thus more years lived with disability [4]. Comorbidity per se predicts many adverse health outcomes, including the onset of additional health problems such as neuropsychiatric symptoms and mental illness. Mood disorders, including depression, are present in up to 50% of these patients, resulting in impaired physical and cognitive functions [5]. It is becoming an urgent need for geriatrics and gerontologists to help older persons stay in good mental and physical health for as long as possible. In this context, psychological well-being represents a key element, considering that mental fitness is also associated with a risk reduction in chronic physical diseases and with the promotion of longevity.

At a biological level, aging is a universal process, mainly characterized by the accumulation of “damage” linked to several mechanisms known as the nine hallmarks of aging [6,7]: genomic instability and telomere attrition, epigenetic alterations, loss of proteostasis, mitochondrial dysfunction, deregulated nutrient sensing, cellular senescence, stem cell exhaustion, and altered intercellular communication. Among these mechanisms, telomere attrition has been considered as the primary hallmark [4]. Interestingly, telomere length (TL), as an indicator of cellular aging, is also tightly connected to psychological stress. It has been well described that a relationship exists between stress, telomere shortening, and mental disorders, even if the nature of this link remains mostly unknown.

This study involved a mini-review of the major findings from human studies investigating the link between psychological wellbeing and successful aging, with a particular focus on telomere dynamics. All human research reported in the English language and published in peer-reviewed scientific journals within the past 15 years was reviewed. The literature search was conducted in MEDLINE using the following keywords: “wellbeing”, “telomere”, “stress”, “stress and aging”, “psychological wellbeing”, “psychological wellbeing and aging”, “successful aging”, “healthy aging”, and “telomere and aging”. The keywords were used in all combinations to retrieve the maximum number of articles.

To provide a short overview, the paper begins with a summary on definitions of healthy aging and psychological wellbeing. Subsequently, special attention is given to studies integrating the contribution of psychological stress to telomere dynamics and healthy aging.

## 2. Defining Healthy Aging

“Every man desires to live long, but no man wishes to be old”, said Jonathan Swift in the 17th century. Notwithstanding numerous theories, aging remains a largely mysterious process. In humans, this process has three main features: (1) universal, (2) progressive, and (3) continuous. Aging is characterized by a dynamic decrease in physiological capacities, which leads to increased vulnerability to diseases and ultimately death. However, this process is not fixed but malleable, and its rate can be accelerated or decelerated at any point in life. Another feature of aging is its extreme heterogeneity among individuals, and three different phenotypes have been described so far: (1) accelerated aging, (2) normal or usual aging, and (3) successful aging, depending on the level of physical, social, and cognitive functions [8]. Defining successful aging is not easy, and there is still a lack of agreement among researchers in the field. In 1998, Rowe and Kahn [8] defined successful aging as a complex concept including properties of both physical and psychological health in old age allowing subjects to maintain a low risk of disease and associated disabilities. Later, Moody and collaborators [9] underlined that the term “successful aging” is related to “key ideas such as life satisfaction, longevity, freedom from disability, mastery, growth, active engagement with life, and independence”. In fact, older age is generally the time towards the end of life—a period during which a person experiences many stress-promoting events, from the decline of physical functions to a reduction in economic power as well as loneliness and related social isolation [10]. Thus, it is difficult to think about older age as a time of health. Instead, other biomedical perspectives have adopted a more psychosocial point of view regarding successful aging, focusing on a strong adaptation to the aging process and associated psychological resources. Therefore, successful aging is also called “healthy aging”, “active aging”, or “productive aging”, which suggests that this later life stage can be a time of vitality and health [9,11]. From this point of view, aging can be considered as the final result of the balance between gains and losses. This perspective and experience of aging are more subjective, and include satisfaction, positive emotions, and good mental health. Thus, from this point of view, aging is not necessarily a burden, as there is a small part of the population that is not disabled in later life. Some centenarians can enjoy a high quality of life and make valuable and significant contributions to society, even with the limitations that inevitably age per se induces.

### 2.1. Stress and Aging

According to various theories, at the biological level, aging is characterized by the accumulation of molecular damage that progressively leads to structural and functional abnormalities in cells, tissues, and systems. In this context, the environment plays a decisive role, and the body’s ability to overcome stress and respond correctly to external insults or stressors represents the primary determinant of longevity [12]. All living organisms experience a wide range of stressors during life [13]. For cells in culture, environmental stressors include any factors that can damage them, such as radiation or osmotic changes. In humans, the term “stress” is often used to refer to actual life events, such as losing a family member. Stress is also used to refer to the biological and emotional reactions that some situations may evoke in a person, and the so-called “stress responses”. Collectively, stress may be considered as a “state of mind”, that involves the brain, the whole body, and their complex connections. This condition is lived differently among individuals, and it may reflect real-life events from minor to major ones that each person may feel differently. Daily stress accumulates progressively along with aging, and it may negatively affect the individual’s health and wellbeing. Typical stressors experienced in the context of aging include chronic illnesses, cognitive impairment, the psychosocial stress of caregiving, or personal losses of people, independence, and financial resources. However, stress is not a unitary construct, but rather comprises the exposure to stressors, the perception of stress, and the physiological stress response. Individuals react very differently to life adversities: some succumb to depression as a result of these adversities, and some continue to lead a life of personal fulfillment despite those restraints.

At the physiological level, acute stress is known to negatively affect neuroendocrine function via the hypothalamic–pituitary–adrenal axis (HPA). The central elements of the physiological stress response are cortisol exposure as well as individual cortisol reactivity. When this feedback is continuously stimulated, it results in the secretion of cortisol during chronic stress. Sustained higher levels of cortisol can lead to a serious health risk. In fact, high levels of chronic stress are associated with many diseases and deleterious conditions, including metabolic syndrome, immune compromise, cardiovascular diseases, and mental disorders [14]. HPA dysregulation has been implicated in several late-life disorders including anxiety, major depression, and cognitive impairment [15]. Nevertheless, evidence from animal studies suggests that stress can be either detrimental or beneficial in the aging process. On the one hand, it is clear that stressors that are chronic and persistent and called “toxic stress” may contribute to disease pathogenesis [12]. Then, it is likewise clear that restricted or reasonable stressors may result in physiological advantages, presumably because of the activation of stress reactions and metabolic changes. This is usually called “hormetic stress”. Hormone release linked to chronic stress may protect the body in the short run by an adaptation induction and so-called *allostasis* or “stability through change”. Circulating catecholamines constitute a key part of allostasis and can have synergistic and oppositional effects on the actions of glucocorticoids [16]. Instead, in the long run, this burden may cause structural and functional changes in the brain and the whole body that can lead to disease susceptibility and defined *allostatic load*. Allostatic load is “the wear and tear on the body” that accumulates as an individual is exposed to repeated or chronic stress. It represents the physiological consequences of chronic exposure to psychological stress, and it is linked to changes in the autonomic, neuroendocrine, metabolic, and immune systems [17,18]. It has also been shown that psychological stress may change limbic and prefrontal cortex areas, which regulate emotional health as well as cognitive function. Chronic daily stress can lead to physical and cognitive disorders that hurt the health and well-being of older adults, exerting a negative influence on successful ageing [19]. Clearly, as with everything in nature, the duration, severity, and perception of a stressor can be salutary or damaging to a person. Psychological “stress resilience” during adversities is thought to be related to a combination of genotypes particularly resistant to stress, temperament, and strong behavior, which in combination can promote greater longevity [17].

### 2.2. Psychological Wellbeing: The Key to Longevity

The epidemiological transition has suggested the importance of promoting physical health and psychosocial well-being among older subjects. According to the “Mental Health Action Plan 2013–2020” [20], mental health is an integral part of health and well-being which includes individual, social, cultural, economic, and environmental factors. Psychological or subjective well-being (WB) is a complex and multifaceted concept where three aspects can be distinguished [20]: (1) the individual’s affective status (e.g., positive or negative emotional states), (2) ability (e.g., adaptive capacity or coping skills), and (3) perception (e.g., satisfaction, purpose, and outlook). Regarding these elements, three types of WB measures have been developed so far: (1) *Evaluative or global well-being*, involving global assessments of how people evaluate their lives or their satisfaction with life. It assesses an overall judgment of individual life, including one’s aspirations, achievements, and current circumstances. (2) *Affective or hedonic well-being*, involving measures of feelings such as happiness, sadness, and enjoyment. It captures affective components of WB, such as the experience of joy or stress. (3) *Eudemonic well-being*, which focuses on judgments about the meaning or purpose of one’s life and appraisals of constructs such as fulfillment, autonomy, and control [18].

Evidence shows an association between global, hedonic, and eudemonic well-being with successful aging. High levels of subjective well-being may boost physical health and longevity as well [21]. It seems that happy people live longer and in health [22]. Accordingly, the English Longitudinal Study of Ageing showed that personal well-being is associated with higher survival rates [23], even if this relation varies among nations [24]. Prospective studies have found that older people with greater wellbeing are less likely to develop declines in activities of daily living [24], show a significantly slower decline in the walk speed [25], and make a better recovery in motor, cognitive, and functional performance, even after a major cardiovascular event [26]. The survival benefit associated with higher well-being is particularly marked in older subjects, as shown in a meta-analysis including over thirty prospective studies where greater positive well-being was linked with a reduced risk of mortality both in healthy and ill populations [27]. Indeed, substantial evidence also supports that some behavioral factors (e.g., avoiding smoking and alcohol consumption, adhering to a healthy diet, participating in physical activity) and social factors (including relationships and socialization) are strongly associated with health and wellbeing, particularly later in life [28,29].

## 3. Telomere Biology at the Heart of Aging

The aging process, from the cellular level to whole-body function, is regulated by a powerful “biological clock”, made by the telomere/telomerase system. Telomeres are a structure with protective functions at the ends of chromosomes that, together with specific proteins, form an end “cap” for genome stability and integrity preservation. They consist of non-coding DNA made of repeats of short sequences of nucleotides rich in guanine. In humans, this DNA is double-stranded for most of its length, with a 3′G rich single-stranded overhang of several repeats at the very end (150–200 bp), called the “G-strand overhang” consisting of TTAGGG repeats. Telomeres need to be long enough to form a T-loop structure in order to function properly. Only when properly structured can telomeres prevent chromosomes’ ends from being wrongly identified as breaks in the double-strand [30]. Because of the inability of DNA polymerase to maintain the length of the 3′ overhang (the so-called “end replication problem”), telomeres lose some nucleotides at each cell division or replication event, becoming shorter and shorter. When telomeres get too short and become dysfunctional, a sustained DNA damage response is activated that leads the cell to stop dividing and become senescent. The ability to restore or lengthen telomeres depends mainly on the action of a ribonucleoprotein enzyme called “telomerase”, which provides a powerful telomere maintenance mechanism [31]. Most human somatic tissues and adult stem cells undergo telomere shortening with aging because they do not express a sufficient amount of telomerase to maintain telomere length indefinitely [32]. This phenomenon is the well-known “Hayflick limit”, and in a few words, it represents the maximum number of divisions that cells may undergo in their lives, and it is determined by the telomere erosion rate. Telomere shortening could also represent the consequence of an alternative mechanism called “telomere uncapping”, which consists of an interference between the telomeric sequence and telomere-binding proteins. Many studies have shown a strong correlation between telomere attrition and aging. In fact, extremely shortened telomeres limit the rate of cell proliferation and are associated with disease risk and mortality [33]. However, it remains unclear how telomeres lead to aging. New evidence is coming out showing that the rate of telomere attrition with age is highly heterogeneous and variable among individuals, and telomeric replication defects other than the end replication problem may contribute to the aging-associated telomere erosion in humans [34]. In fact, due to their nature (i.e., they are constituted of G-rich repetition) and structures, telomeres are regions that are very difficult to replicate. Following DNA replication stress, replication forks can stall and terminate before telomeres, leaving some chromosome ends incompletely replicated. This can cause cells to undergo senescence prematurely, even when telomeres are long and not critically short. Thus, new evidence suggests that dysfunctional telomeres lead to premature cellular senescence regardless of whether they are too short or long, highlighting the necessity to analyze the state of each telomere in every single cell and not measuring the total telomere lengths (TLs) on average [34].

### 3.1. Telomere Length: The Hypothetical “Meter” of Aging

Telomere length is mainly measured in white blood cells, indicated as leukocyte TL (LTL). Recently, a “telomeric brink” hypothesis has been postulated [35], which states a causal role for telomere shortening in longevity modulation: very critically short telomeres increase the risk of death [30]. Again, animal species with shorter mean telomere lengths at birth or with faster telomere shortening have limited lifespan as compared with counterparts. In humans, an LTL of 5 kb denotes a high risk of imminent death. Accordingly, several studies have shown that shorter LTL is usually associated with an increased risk of age-related diseases and accelerated aging phenotypes [36]. More recently, some exceptions have also been reported, such as the association of longer telomeres with an increased risk of cancer susceptibility (in breast, lung, pancreas, or melanoma) as well as some age-related diseases including cognitive impairment [37]. These observations suggest that both critically short and dysfunctional longer telomeres might be considered as “hallmarks” of aging. This assumption may also prove the huge inter-individual and intra-individual variability found among people of the same age, which made LTL a questionable marker of aging. It also underlines that chronological age is a relatively imprecise measure of an individual’s health status. LTL is influenced by many factors, including age, gender, and race. Instead, the association to other factors, such as smoking habit, alcohol consumption, physical activity, diet, socioeconomic status and education, body mass index, markers of glucose metabolism, and blood pressure is inconsistent across studies [38]. Telomere length balance may be considered as the best indicator of an individual’s age: this finely regulated balance is a function of somatic growth rate, genetics and epigenetic factors, age itself, physical illness, environment (including pollutants), and behavior (including unhealthy diet, obesity, smoking, lack of physical activity, and sleep disorders) [39]. More evidence supports the notion that psychosocial conditions can also impact TL [40]. Physiologic context, psychological response to the stress at the individual level, and mental illness are all involved in the rate of change in mean TL at any given time. Psychosocial factors (including acute and chronic psychological stress, maternal stress, childhood maltreatment, trauma, or major depressive disorders) are associated with accelerated telomere attrition during life [41], even if the evidence of a direct association is still lacking.

### 3.2. Centenarians: Slow Telomere Attrition in the Escaper Phenotype?

Looking at the distinctive characteristic of long-lived subjects may be the key to discovering the elixir of slow aging. Three different subgroups of the oldest old can be described, expressing different pathways of aging: (i) the *escapers*, (ii) the *delayers*, and (iii) the *survivors*. The escapers are those selected people who reach the extreme age, 100 years and more, without diseases; the delayers, those who start to be affected by chronic diseases after 85 years old; and finally the survivors, who survive together with their chronic diseases after 85 years old. Thus, the “oldest old” is an extremely heterogeneous group. The oldest old must also be differentiated from the “young old” (65–74 years) and the “old” populations (75–84 years) because they represent a distinct clinical and pathological entity. Studies on telomere dynamics in long-lived persons or longitudinal studies are still few. Collectively, escaper centenarians who have generally escaped major age-related diseases have been found to have longer telomeres [42,43] as well as better maintenance of telomere length [44]. Thus, variations or polymorphisms in the human telomerase gene that are associated with better maintenance of telomere length may confer healthy aging and exceptional longevity in humans. Telomere length is greater in the escaper centenarians as compared with low-performing centenarians [45]. Additionally, telomerase activity in white blood cells after in vitro stimulation is higher in the high-performing centenarians [45]. Why escaper centenarians age differently is unknown. Having long telomeres or slower telomere attrition during life might be important factors linked to longevity [44]. Interestingly, even though centenarians are more prone to chronic stress, more than half of our centenarians are not depressed [39]. They collectively show an emotional behavior to life events and contrast stressful conditions with low anxiety [46].

## 4. Psychological Stress and Telomere Dynamics

A shorter LTL is strongly associated with psychological stress as well as several mental disorders, including depression [47]. Perceived chronic stress, as well as a stressful life, have been associated with shorter telomere length and an altered telomere maintenance system. Stress, as experienced and lived in depression, leads to physiological changes, which in turn accelerates telomere attrition [47]. Everything seems to start at birth, or even before in the maternal uterus. It has also been suggested that prenatal adversities influence newborn mean telomere length. In fact, newborns whose mothers experienced a high level of stress during pregnancy had significantly shorter telomere length at birth as compared with newborns of mothers with lower or no perceived stress [48]. Early childhood adversity or trauma (e.g., sexual abuse, neglect, exposure to any kind of violence) is associated with greater telomere attrition [49]. During adulthood, stressed, depressed, and anxious subjects show shorter telomeres as compared with psychologically healthy counterparts [50,51].

Shortened telomere length and decreased telomerase activity have been associated with psychological and life stress in many studies. Some of the first evidence that psychosocial stress might influence telomere dynamics was the results from a study conducted by Epel and collaborators in 2004 [52] comparing stressed mothers caring for their chronically ill kids and low-stress mothers with a healthy child. Mothers with higher perceived stress had shorter telomeres as well as lower telomerase activity than mothers with lower perceived stress. In a follow-up study [53], the same research group found that reduced telomere length correlated with increased nocturnal urinary cortisol and catecholamine levels, while low telomerase activity correlated with increased nocturnal urinary epinephrine and greater decreases in heart rate variability during a laboratory procedure used to reliably induce stress in human research participants—the Trier Social Stress Test (TSST). Subsequent studies have examined telomere length and telomerase activity in various stress-related contexts. Damjanovic et al. [54] reported shorter telomeres and increased telomerase activity in caregivers of subjects affected by Alzheimer’s disease as compared with controls. Kiefer and colleagues [55] observed reduced telomere length in women with greater dietary restraint, defined as a chronic preoccupation with weight and chronic psychological stress. In an epidemiological study of 647 sisters of women affected by breast cancer, Parks and collaborators [56] found that reduced LTL correlated with perceived stress, especially in women who were older, had a recent major loss, or had higher morning urinary epinephrine levels. In a small study of female caregivers of dementia partners and controls [57], it has been found that shorter telomeres are associated with greater salivary cortisol responses to the TSST and higher overnight urinary free cortisol. In an expanded larger sample from the same study, telomerase activity was lower at baseline in caregivers but rose in both groups during the TSST [58]. Indeed, in another small study conducted in a cohort of dementia caregivers, it has been shown that even a brief daily meditation activity can lead to an improved mental function and reduced levels of depressive symptoms, associated with a significant increase in telomerase activity over few months [59]. Together, these studies suggest that sustained stress results in accelerated cellular aging.

Many are the identified mechanisms underlying the association between stress and telomere dynamics. One of the models proposed to explain this relationship is the deregulated allostatic load model. This model proposes that chronic stress influences the regulation of the HPA axis by increasing cortisol secretions, contributing to allostatic load and in turn impaired telomere maintenance [57]. In fact, in humans, shorter telomeres are associated with greater cortisol reactivity [60] and evidence in vitro suggests that high glucocorticoid levels correlated with lower telomerase activity [61]. Adults with major depressive disease tend to have shorter telomere length [54], especially with greater severity and duration of depression [62]. Results on telomerase may seem contradictory. Nevertheless, it is important to note that under conditions of acute stress there is first a depletion of circulating immune cells and thus a compensatory increase of young cells, resulting in longer telomere determination as well as higher telomerase activity. Instead, chronic stress induces sustained replication stress and in turn accelerated telomere attrition, as well as the reduction in telomerase activity. This aspect could explain the “telomerase paradox”: during acute stress, telomerase is more activated to protect telomeres; while in chronic stress, as seen in depressed subjects, the activity may be lower, which leads to progressive telomere dysfunction [63]. A stress triad on telomere maintenance has also been hypothesized and described: chronic major stressor exposures lead to persistently high levels of perceived stress and subsequent stress arousal, which in turn significantly impact telomere attrition [64]. Other studies further suggest that inflammation significantly contributes to telomere attrition and that sustained stress is linked to low-grade inflammation and shorter telomeres compared to healthy people, as recently reviewed in [40].

## 5. Conclusions and Final Remarks

Telomere attrition is accelerated under conditions of chronic stress and stressful events during life. Long-lived subjects, such as centenarians, have a lower incidence of depressive symptoms and other mental problems, suggesting that their personality and behavior may be the secret for longevity promotion. Moreover, centenarians may have slower telomere attrition during life that might be linked to longevity. Well-being in old-age subjects remains an essential determinant of longevity, and there is suggestive evidence that global positive well-being is relevant to health and quality of life along with aging. However, research into psychological well-being and healthy aging and in particular in older subjects are at an early stage. Although evidence has reported promising results related to the telomere/telomerase system and psychosocial factors, there are several methodological weaknesses among studies. Many of the studies are retrospective, with cross-sectional design and small sample size, making it difficult to find a causal association. There is substantial variability in LTL in humans due to many intrinsic and extrinsic factors. Therefore, large samples are required to increase the potential generalizability of reported and suggested effects. Future studies would benefit from large-scale randomized controlled trials, and where possible, longitudinal design with LTL and telomerase activity measurement. Most importantly, health care systems should support strategies for improving positive mental states during all life stages. Much of our knowledge about well-being and psychological fitness at older ages is the result of small-population cohort studies, and more interest and investment in this research field is essential. Novel methods or scales to assess hedonic well-being need to be developed, as does our understanding of linking mechanisms between positive psychological states, healthy aging, and telomere dynamics.
